# Abnormal Dorsal Caudate Activation Mediated Impaired Cognitive Flexibility in Mild Traumatic Brain Injury

**DOI:** 10.3390/jcm11092484

**Published:** 2022-04-28

**Authors:** Hui Xu, Xiuping Zhang, Guanghui Bai

**Affiliations:** 1 Department of Radiology, The Second Affiliated Hospital and Yuying Children’s Hospital of Wenzhou Medical University, Wenzhou 325027, China; 2 Research Center, Institut Universitaire de Gériatrie de Montréal, Montreal, QC H3W 1W5, Canada; 3 School of Psychology, Beijing Language and Culture University, Beijing 100083, China; zhangxp@blcu.edu.cn; 4 Wenzhou Key Laboratory of Basic Science and Translational Research of Radiation Oncology, Wenzhou 325027, China

**Keywords:** mild traumatic brain injury, functional MRI, cognitive flexibility, task switching, dorsal caudate

## Abstract

Background: Mild traumatic brain injury (mTBI) is an important but less recognized public health concern. Previous studies have demonstrated that patients with mTBI have impaired executive function, which disrupts the performance of daily activities. Few studies have investigated neural mechanisms of cognitive flexibility in mTBI patients using objective tools such as the psychological experiment paradigm. Here, we aimed to examine neural correlates of cognitive flexibility in mTBI. Methods: Sixteen mTBI patients and seventeen matched healthy controls (HCs) underwent functional MRI during a rule-based task-switching experimental paradigm. Linear models were used to obtain within-group activation maps and areas of differential activation between the groups. In addition, we conducted mediation analyses to evaluate the indirect effect of abnormal dorsal caudate activation on the association between information processing speed and cognitive flexibility in mTBI. Results: mTBI patients exhibited significantly longer reaction time in the task switching (TS) condition compared to HCs, reflecting impaired cognitive flexibility. In addition, the patients showed reduced activation in the dorsal caudate (dCau), anterior cingulate cortex, and other frontal regions during the TS condition. Mediation analysis revealed that the reduced dCau activation had a significant effect on the relationship between information processing speed and cognitive flexibility in mTBI. Conclusions: Abnormal dorsal caudate activation in mTBI mediates impaired cognitive flexibility, which indicated dorsal caudate might be playing a vital role in the cognitive flexibility of mTBI patients. These findings highlight an alternative target for clinical interventions for the improvement of cognitive functions in mTBI.

## 1. Introduction

Mild traumatic brain injury (mTBI) is an important but less recognized public health concern [1], which accounts for nearly 80% of all traumatic brain injuries [2,3]. Patients with mTBI have impaired executive function, which disrupts the normal performance of daily activities [4]. Executive function is a high-level cognitive function in human beings [5]. As a core component of the executive function, cognitive flexibility defines the ability of individuals to constantly adjust their behavioral responses according to the changing external environment [6,7]. Many studies have demonstrated executive dysfunction in patients with various diseases and the patients exhibit impaired cognitive flexibility [8,9,10,11,12].

Previous studies conducting magnetic resonance imaging (MRI) found that patients with traumatic brain injury have executive dysfunction, and showed impaired cognitive flexibility and altered information processing speed on the behavior level [13]. A previous study that employed graph theory analysis showed that patients exhibited reduced centrality of characteristic vectors of caudate and cingulate cortex, which could accurately predict executive dysfunction in traumatic brain injury [13]. Another study that conducted a word-based working memory task in patients with severe traumatic brain injury showed that the abnormal activation response of caudate was negatively correlated with the patient’s cognitive fatigue under complex conditions, but was positively associated with cognitive fatigue under simple conditions [14]. In addition, one task-switching study showed that patients with local caudate atrophy had abnormal cognitive flexibility, and needed sufficient cognitive load during task switching, but exhibited a significantly increased error rate [15]. 

Besides, other studies demonstrated that patients with traumatic brain injury had widespread altered white matter microstructure, and the damage to the upper radiation crown from the caudate to the anterior auxiliary motor zone was significantly associated with the switching cost required for patients to achieve task switching, which could predict impaired cognitive flexibility in the patients [16]. Therefore, patients with traumatic brain injury exhibited impaired cognitive flexibility with altered behavioral responses as well as structural and functional abnormalities in the hub region caudate. However, these studies mainly focused on the cognitive dysfunction in patients with moderate to severe traumatic brain injury.

Moreover, previous studies mostly employed specific neuropsychological measurement tools such as the Wisconsin Card Sorting Test to assess cognitive function [17], but were limited by subjective judgments and the expectation effect; it has been proved that when individuals with mild head injury are informed of this, they may experience cognitive difficulties and perform worse on neuropsychological tests compared to the individuals who are uninformed [18,19]. In addition, other studies conducted different psychological experimental tasks to measure cognitive functions in patients with traumatic brain injury, such as working memory tasks [20,21,22] and the go/no-go task [23,24], which were performed mainly to measure working memory and inhibitory control ability, respectively. Furthermore, few studies have investigated cognitive flexibility in mTBI patients and its associated neural mechanisms using objective tools such as the psychological experiment paradigm.

To explore underlying neural mechanisms of cognitive flexibility in mTBI patients, we performed a rule-based cognitive control experimental paradigm with functional MRI. Here, we first investigated specific behavioral patterns of cognitive impairment in the mTBI patients, and then explored abnormal brain activation in task conditions. Thereafter, we assessed the relationship between altered brain activation, information processing speed and cognitive flexibility. We aimed to identify neural correlates of impaired cognitive flexibility in mTBI.

## 2. Materials and Methods

### 2.1. Participants

Sixteen mTBI patients (6 females, mean age 25.8 ± 2.8 years) were recruited from the local emergency department. Diagnosis of the mTBI was assessed by two experienced neurologists following the World Health Organization’s Collaborating Centre for Neurotrauma Task Force [4]. To be included in this study, all mTBI patients had to have met the following inclusion criteria: (1) a Glasgow Coma Scale score of 13–15; (2) one or more of loss of consciousness (if present) <30 min, post-traumatic amnesia (if present) <24 h, and/or other transient neurological abnormalities such as focal signs, seizure, and intracranial lesion not necessitating surgery. We excluded patients with a history of neurological disease, long-standing psychiatric condition, head injury, substance or alcohol abuse, clinical symptoms of depression and anxiety, intubation and/or presence of a skull fracture as well as administration of sedatives on arrival in the emergency department, spinal cord injury. Patients with a manifestation of mTBI due to medications by other injuries (e.g., systemic injuries, facial injuries, or intubation) or other sources such as psychological trauma, language barrier, or coexisting medical conditions as well as those caused by penetrating craniocerebral injury were also excluded from this study. In addition, 17 age- and sex-matched healthy controls (HCs) were also enrolled (5 females, mean age 27.8 ± 3.3 years). 

### 2.2. Neuropsychological Tests

Several neuropsychological performance tests were performed. The tests included Trail-Making Test Part-A (TMT-A) for rote memory assessment, Forward Digit Span (FDS) and Backward Digit Span (BDS) test of Wechsler Adult Intelligence Scale-III for working memory assessment and Digit Symbol Coding (DSC) task for cognitive function assessment and information processing speed. On the other hand, we employed self-reported symptomatology assessments such as Insomnia, which was evaluated using the Insomnia Severity Index (ISI) for sleep quality and short-form Headache Impact Test (HIT) for severity of headaches. All the neuropsychological tests were performed by an experienced clinical psychologist blinded to this study.

### 2.3. Experimental Design and Procedures

**Figure 1:**
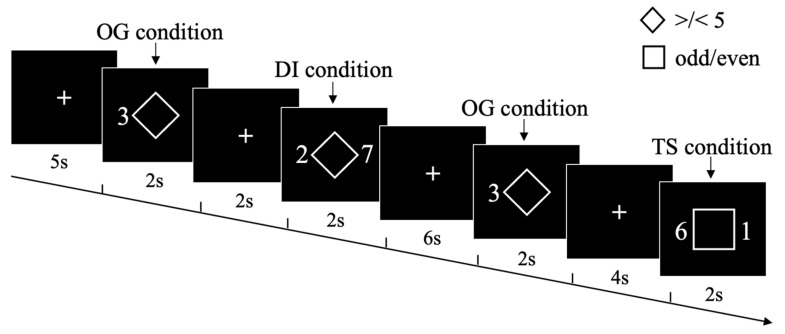
Schematic illustration of the rule-based task-switching experimental paradigm. During three task conditions [ongoing (OG) condition, distractor inhibition (DI) condition, task switch (TS) condition], depending on task cues (square vs. diamond), participants performed one of the different tasks on visually presented number stimuli (smaller/larger than 5 vs. odd/even).

We employed a modified version of the rule-based task-switching experimental paradigm [25,26,27], where participants responded to the target digital stimuli based on the cues presented initially. It is a highly time-efficient event-related fMRI paradigm, which has been designed to specifically probe for cognitive flexibility and stability. Participants were instructed to perform one of three task conditions on numerical stimuli based on a cue presented simultaneously during each trial (Figure 1). The task was generated and presented using PsychToolBox and appeared on a uniform black background [28,29]. At each trial, participants were cued explicitly (using a square or diamond cue) as to which condition should be performed during the digit stimuli between 1 and 9 (excluding number 5). Three conditions were set in the experiment: ongoing (OG), distractor inhibition (DI) and task switch (TS). For the ongoing (OG) condition, a diamond cue was presented at the center of a screen with a digit stimulus on the left side, and participants were asked to indicate whether the digit was larger or smaller than five. On the other hand, for the distractor inhibition (DI) condition, a diamond cue was presented at the center of a screen with two-digit stimuli on each side, and participants were asked to indicate whether the left digit was larger or smaller than five, and had to inhibit their response to the right digit (assessing cognitive stability). For the task switch (TS) condition, a square cue was presented at the center of a screen with two-digit stimuli at each side, and the participants were asked to switch from the left digit to the right digit and then decide whether the right digit was odd or even (assessing cognitive flexibility). At each trial, the condition cue and digit stimuli were simultaneously presented for 2000 ms, followed by a variable inter-trial interval of 2000, 4000, or 6000 ms. Participants were required to accurately respond as quickly as possible within the limit of 2000 ms.

All participants received out-of-scanner practice with trial-to-trial feedback and were instructed to provide accurate and quick responses until they attained 95% accuracy. The task was split into two functional scanning runs of 84 trials each. Each run started and ended with two dummy scans (5 s) for scanner signal stabilization, while the participants looked at a fixation cross at the center of the screen.

### 2.4. fMRI Data Acquisition

Functional MRI images were acquired on a 3.0 Tesla MRI scanner (GE 750 Medical Systems), equipped with a single-shot, gradient-recalled echo planar imaging (EPI) sequence and a 32-channel head coil.

A total of 340 functional volumes were acquired in two runs, using a T2*-weighted BOLD-sensitive gradient-recalled, EPI sequence with 54 slices covering the whole brain [repetition time (TR) = 2500 ms, echo time (TE) = 30 ms, slice thickness = 3 mm, flip angle (FA) = 90°, field of view (FOV) = 216 mm × 216 mm, matrix size = 64 × 64, voxel size = 3 mm × 3 mm × 3 mm]. The first and last two volumes of each run were discarded to allow for stable magnetization.

Before performing functional MRI scan, a high-resolution T1-weighted magnetization prepared-rapid gradient echo scan was acquired with the following parameters: TR = 2300 ms, TE = 3.17 ms, FA = 9°, slice thickness = 1 mm, FOV = 256 mm × 256 mm, matrix size = 256 × 256.

We also captured multiple neuroimaging data (including T1-flair, T2-flair, T2, susceptibility-weighted imaging (SWI)) for all the mTBI patients. These data were used to assess the presence of focal lesions and cerebral microbleeds. Visible contusion lesions were not detected in any of the patients.

### 2.5. Statistical Analysis of Behavioral Data

All trials with a response time (RT) < 150 ms were eliminated. The RT analyses were limited to proper trials. Both RT and error rate (ER) data for the task behavioral performance were analyzed using ANOVAs, with “task condition” (OG, DI, TS) and “group” (mTBI patients, HCs) factors. The significant main effects and interactions were further explored by *post hoc t* tests using Bonferroni’s correction. A *p* <0.05 was used as statistically significant for all the behavior analyses.

### 2.6. fMRI Data Preprocessing and Statistical Analysis

Using FEAT in FSL v5.0, we analyzed whole-brain voxel-wise activation on the fMRI scans of the rule-based task-switching experimental paradigm [30]. We performed preprocessing of all individual fMRI runs using the following procedures: both the first and last 2 volumes were deleted, underwent motion correction (MCFLIRT) [31], brain extraction, spatial smoothing (6-mm full-width at half maximum kernel), and high-pass temporal filtering (0.01-Hz cutoff). In addition, linear registration (FLIRT) was performed in T1, fMRI, and MNI152 standard space. The degree of head motion from all participants was quantified, and according to the head motion criteria (translational or rotational motion parameters less than 2.0 mm or 2.0°), none of the participants were excluded (the mean translation was 0.08 ± 0.02 mm, and rotation was 0.04 ± 0.01°).

First-level (within-run) general linear model analyses in native fMRI space were conducted with FILM prewhitening, with 3 separate regressors (the onset of all stimuli for OG condition, DI condition and TS condition). Each one of them convolved with a double-gamma hemodynamic response function with the application of temporal filtering [32]. First-level contrast was set up to create voxel-wise contrast of parameter estimate maps of activation in different task conditions. The maps were then used for second-level (within-subject) analysis in the 2 runs. The maps were converted to MNI152 space and fixed effects analyses were performed with 3 contrasts to identify OG, DI or TS conditions. The resultant maps for each contrast were then used for third-level (group-level) FLAME 1+2 mixed-effects analyses which proved to have high statistic values at a small proportion of voxels in a relatively small sample size (threshold: cluster-based *p* < 0.05; whole-brain family-wise-error corrected Z > 2.3) [33], with 3 contrasts: OG, DI or TS activation. We employed Student’s *t* test to analyze the effect of the group on brain activation of different task conditions using mTBI patients and HCs as factors.

### 2.7. Relationship between Abnormal Activation, Behavioral and Neuropsychological Measures in mTBI

To examine whether abnormal activation during different task conditions correlates with behavioral features and neuropsychological measures in mTBI, Spearman correlation analyses between abnormal activation, task behavioral performance and clinical parameters were performed in patients. The *p* < 0.05 was taken as a significant threshold with FDR (false discovery rate) corrected for multiple comparisons.

### 2.8. Mediation Analysis between Abnormal Dorsal Caudate Activation and Cognitive Flexibility in mTBI

To examine the indirect effect of abnormal dorsal caudate activation on the association between information processing speed and cognitive flexibility in mTBI patients, we conducted mediation analysis using SPSS PROCESS v3.4, with a 5000 bias-correction bootstrapping approach [34,35]. Here, the information processing speed (indexed by the scores of DSC task) was considered as the independent variable, abnormal dorsal caudate activation in the TS condition was considered as the mediator while cognitive flexibility (indexed by the RT of TS condition) was considered as the dependent variable. Age and education level were used as covariates in the mediation analysis. The estimation of indirect effects was considered significant when zero was not included in the bootstrapped 95% confidence interval (CI) (5000 iterations).

## 3. Results

### 3.1. Demographics and Neuropsychological Assessment

Our analysis showed that there were no significant differences in age (t_31_ = −0.952, *p* > 0.05), education level (t_31_ = −1.297, *p* > 0.05) or sex (χ^2^_1_ = 0.243, *p* > 0.05) between groups. The mTBI patients presented significantly worse insomnia severity and headache compared to HCs (all *p* < 0.01). In addition, patients exhibited impaired information processing speed (reflected by the DSC task) compared with HCs (*p* < 0.001, Table 1). All mTBI patients exhibited the same injury severity with a Glasgow Coma Score of 15, loss of consciousness < 30 min, and post-traumatic amnesia < 24 h. The most common cause of injuries was acceleration/deceleration caused by traffic accidents (6/16, 37.5%), followed by falls (5/16, 31.2%), assaults (3/16, 18.8%), and others (2/16, 12.5%).

### 3.2. fMRI Behavioral Performance

**Figure 2:**
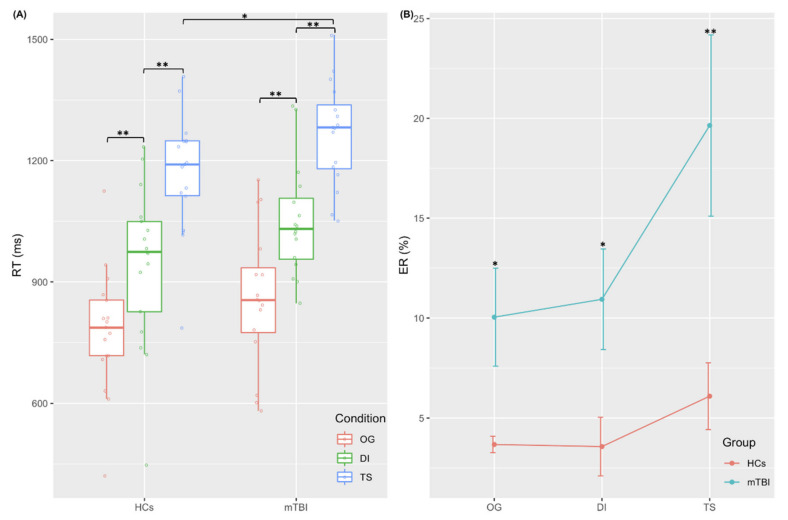
Behavioral results of fMRI task performance. (**A**) Mean response time (RT) during three conditions [ongoing (OG) condition, distractor inhibition (DI) condition, task switch (TS) condition] in mTBI patients and HCs; (**B**) Error rate (ER) during three conditions in mTBI patients and HCs. Error bars represent standard deviation. *: *p* < 0.05; **: *p* < 0.01. mTBI, mild traumatic brain injury; HCs, healthy controls.

Compared with HCs, patients with mTBI exhibited significantly longer RT in the TS condition [F_(1,31)_ =4.247, *p* = 0.048], reflecting impaired cognitive flexibility on a behavioral level (Figure 2A). Furthermore, mTBI patients were significantly less accurate than HCs across all conditions (*p* < 0.05, Figure 2B). The detailed information about fMRI behavioral performance is described in the Supplementary Materials.

### 3.3. fMRI Brain Imaging Results

Compared with HCs, mTBI patients showed significantly reduced activation in widespread frontal regions across OG conditions (Table S1), which were mainly distributed in the medial superior frontal gyrus (mSFG), dorsolateral superior frontal gyrus (dlSFG), medial orbital gyrus (mOG), ventrolateral middle frontal gyrus (vlMFG) and rostrodorsal supramarginal gyrus (rdSG).

During the DI condition, mTBI patients exhibited significantly reduced activation in a broad cingulate-frontal network compared to HCs (Table S2), which included the posterior cingulate cortex (PCC), posterior insula (pIns) and other frontal regions. 

**Figure 3:**
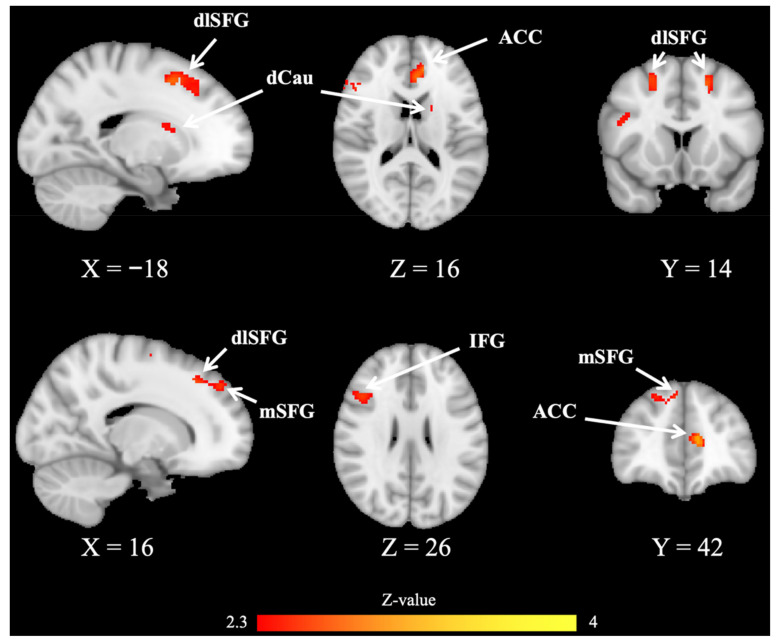
Brain activation results from the BOLD-fMRI analysis of the contrast mTBI < HC in task switching (TS) condition (FEW corrected). Compared with HCs, mTBI patients showed significantly reduced activation in the dorsal caudate (dCau), anterior cingulate cortex (ACC), medial superior frontal gyrus (mSFG), dorsolateral superior frontal gyrus (dlSFG).

In addition, for the TS condition, the mTBI patients showed significantly reduced activation in the dorsal caudate (dCau), anterior cingulate cortex (ACC), and other frontal regions (Figure 3, Table 2) compared with HCs.

### 3.4. Relationship between Abnormal Activation, Behavioral and Neuropsychological Measures in mTBI

The reduced dCau activation of the TS condition in mTBI patients was positively correlated with information processing speed (indexed by DSC) (*p* < 0.001, FDR corrected), but negatively correlated with RT for TS condition (*p* < 0.001, FDR corrected). In addition, the RT for TS condition was negatively correlated with information processing speed in mTBI patients (*p* < 0.001, FDR corrected).

### 3.5. Abnormal Dorsal Caudate Activation Mediates the Relationship between Information Processing Speed and Cognitive Flexibility in mTBI

**Figure 4:**
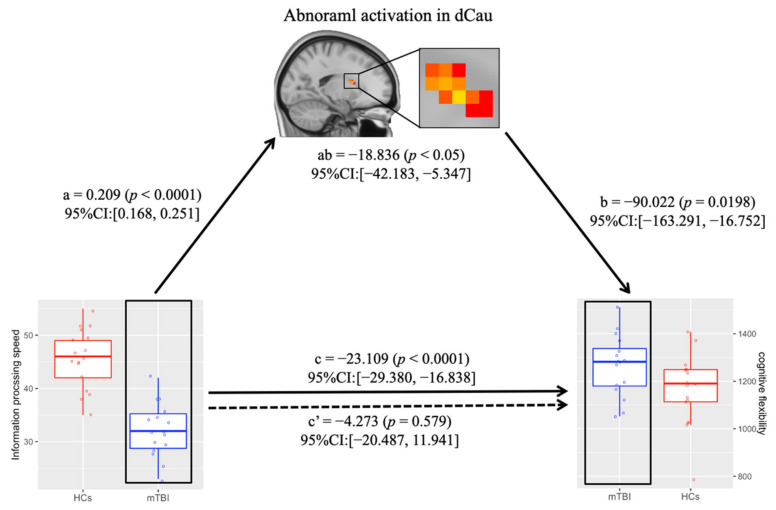
Abnormal dCau activation mediates the association between information processing speed and cognitive flexibility in mTBI patients. The illustration demonstrates that within mTBI patients, information processing speed affected cognitive flexibility through the abnormal dCau activation. mTBI, mild traumatic brain injury; HCs, healthy controls; dCau, dorsal caudate; CI, confidence interval.

Mediation modeling was performed to test whether the magnitude of information about processing speed’s effect on cognitive flexibility was dependent on reduced dCau activation. We found that reduced dCau activation had a significant mediating effect on the relationship between information processing speed and cognitive flexibility in mTBI patients (a ×b = −18.836, 95%CI: [−42.183, −5.347], *p* < 0.05, Figure 4).

## 4. Discussion

To the best of our knowledge, this is the first study to investigate the underlying mechanisms of cognitive flexibility in mTBI patients using a rule-based task-switching experimental paradigm. The mTBI patients exhibited a longer RT than HCs on the TS condition, accompanied by a significantly reduced activation in the dorsal caudate, anterior cingulate cortex and other frontal regions. Furthermore, the abnormal dorsal caudate activation mediated the relationship between information processing speed and cognitive flexibility in patients. Together, these results suggested that dorsal caudate might play a vital role in the cognitive flexibility of mTBI, thus providing an alternative clinical target for impaired cognitive functions in patients.

During the task behavioral performance, mTBI patients only showed significantly longer RT on TS condition compared to HCs, indicating that mTBI patients had impaired cognitive flexibility on the behavior level. These data were in sync with data from previous studies [15,16]. These studies used local-global task-switching tasks and showed that, compared with HCs, patients with traumatic brain injury had significantly longer RT and increased ER under TS condition. Although the studies performed different experimental paradigms, the findings demonstrated specific cognitive behavioral patterns in patients with traumatic brain injuries. Previous research found that patients with several neurocognitive disorders exhibited poor cognitive outcomes [36,37]. We speculated that the mTBI patients had specific behavioral response patterns on impaired cognitive flexibility, which could help in understanding cognitive dysfunction in mTBI.

By comparing the brain activation response of mTBI patients and HCs under different task conditions, we showed that during the OG condition, mTBI patients exhibited significantly weaker activation in widespread frontal brain regions. Plenty of MRI studies revealed that the frontal cortex is involved in different neural circuits with a subcortical nucleus, which plays a vital role in various kinds of cognitive functions [38,39,40,41]. Many clinical studies have demonstrated that mTBI patients often present significantly reduced activation in the frontal cortex during simple tasks [42,43]. On the other hand, the observed abnormal frontal activation in mTBI indicated that the patients had cognitive dysfunction. In addition, during the DI condition, mTBI patients showed significantly reduced activation in the PCC, pIns and other frontal regions. As a key hub in the default mode network, PCC is involved in the regulation of attention and self-referential processing [44,45,46]. It has been hypothesized that reduced PCC activation in mTBI reduces the ability of other stimuli to direct patients’ attention away from the distract stimulus, as patients showed significantly longer RT for the DI condition compared with the OG condition.

In the TS condition, our analyses showed that patients had significantly longer RT and higher ER compared to HCs, thus reflecting impaired cognitive flexibility in mTBI. Furthermore, compared with HCs, mTBI patients exhibited reduced activation in dCau, ACC and other frontal regions. Previous studies showed that the ACC, a hub region in the salience network [47], plays an important role in cognitive control [23], and has a close projection loop with the caudate nucleus [48,49]. Several studies performed the Stroop task, and showed that conflicts between potential behaviors elicit a significant abnormal activation during conflict monitoring and action selection [50,51,52,53]. In addition, the interconnection of the caudate nucleus and ACC is involved in evaluating the consequences of behavior and adjusting behavioral response [53,54,55]. Previous studies have shown that the caudate nucleus is a susceptible hub region in mTBI [56], and its dysfunction was significantly associated with impaired cognitive function in patients [57]. In addition, the impaired white matter fiber from the caudate nucleus to the cerebral cortex could predict impairment of cognitive flexibility in mTBI patients [16]. In our study, mTBI patients might have altered white matter connections in the dorsal caudate, which resulted in reduced activation in the patients. Another study showed that the abnormal caudate activation in patients with traumatic brain injuries was significantly associated with cognitive fatigue [14]. Our results suggested that mTBI patients might need more cognitive load to perform cognitive flexibility responses, which would further cause cognitive fatigue, and finally lead to the abnormal activation of dorsal caudate [58]. 

Interestingly, we demonstrated that the abnormal activation of dorsal caudate played a significant mediating role between information processing speed and cognitive flexibility in mTBI patients. Many previous studies on traumatic brain injury have shown that structural and functional abnormalities of the caudate nucleus were significantly related to executive dysfunction in patients [14,57]. Besides, we showed that information processing speed was significantly related to cognitive flexibility in mTBI patients. This was consistent with the findings from previous studies, which showed that an individual’s information processing speed ability is significantly associated with cognitive flexibility [59,60]. However, whether the impaired information processing speed or the abnormal dorsal caudate activation affects cognitive flexibility in mTBI patients remains unknown. Investigating the relationship between the information processing speed, abnormal dorsal caudate activation, and cognitive flexibility would help to understand the neural mechanisms of cognitive flexibility deficit in mTBI patients. Our mediation model showed that the abnormal dorsal caudate activation was a significant mediator between the patient’s information processing speed and impaired cognitive flexibility.

There are some limitations that should be noticed. Firstly, it is still unclear whether the observed abnormal activation changes could be related to structural and resting-state functional alterations in mTBI patients. Future studies can combine multimodal MRI imaging to investigate the neural mechanism underlying cognitive flexibility in mTBI. Secondly, the difficulty level of the experimental task in this study was relatively high for patients. Thus, future studies can apply cognitive tasks with a moderately difficult level to investigate cognitive function in mTBI. Finally, the sample size of both groups was relatively small. Future studies with larger samples are needed to improve the reliability of the results and investigate the effect of different brain injury types on results.

## 5. Conclusions

In summary, we performed a rule-based task-switching paradigm to investigate the neural mechanism of impaired cognitive flexibility in mTBI. The mTBI patients showed reduced activation in the dorsal caudate, anterior cingulate cortex, and other frontal regions during the TS condition. Further mediation analysis revealed that impaired cognitive flexibility was mediated by abnormal dorsal caudate activation in mTBI. These findings may underscore the importance of dorsal caudate in impaired cognitive flexibility in mTBI, and abnormal dorsal caudate activation reflected in altered cognitive flexibility behavior in patients. Furthermore, our findings would provide an alternative target for clinical interventions for cognitive function improvement in mTBI. In future research, cognitive intervention together with transcranial magnetic stimulation targeted caudate or caudate-involved functional networks will be a great treatment intervention for cognitive impairment in mTBI patients.

## Figures and Tables

**Table 1:** Demographic characteristics and neuropsychological measures in mTBI patients and HCs.

	mTBI Patients (*n* = 16)	HCs (*n* = 17)	t/χ^2^ Test	*p*-Value
Demographic characteristics				
Age (years)	25.8 ± 2.8	27.8 ± 3.3	−1.897	0.067
Sex (F/M)	6/10	5/12	0.243	0.622
Handedness (L/R)	0/16	0/17		
Education level (years)	13.9 ± 1.3	13.6 ± 1.6	0.316	0.754
Time post injury (days)	2.7 ± 1.3	-	-	-
Injury severity (*n*(%))				
GCS = 15	16(100%)	-	-	-
loss of conscious < 30 min	16(100%)	-	-	-
post-traumatic amnesia < 24 h	16(100%)	-	-	-
Injury causes (*n*(%))				
Traffic accident	6(37.5%)	-	-	-
Fall	5(31.2%)	-	-	-
Assault	3(18.8%)	-	-	-
Others	2(12.5%)	-	-	-
Information processing speed				
TMA-A score	50.0 ± 8.8	48.6 ± 8.1	0.461	0.648
DSC score	32.2 ± 5.1	45.7 ± 5.5	−7.343	*p* < 0.001
Working memory				
FDS score	8.5 ± 0.8	7.9 ± 1.1	1.874	0.07
BDS score	4.3 ± 0.8	3.8 ± 0.8	2.036	0.051
Self-reported symptom				
ISI score	7.1 ± 2.1	2.1 ± 1.1	8.704	*p* < 0.01
HIT score	48.1 ± 4.3	38.6 ± 6.1	5.091	*p* < 0.01

Values presented as Mean ± SD unless otherwise stated. mTBI, mild traumatic brain injury; HCs, healthy controls; GCS, Glasgow Coma Score; TMT-A, Trail-Making Test Part-A; FDS, Forward Digit Span; BDS, Backward Digit Span; DSC, Digit Symbol Coding; ISI, the Insomnia Severity Index; HIT, the short-form Headache Impact Test.

**Table 2:** Results from the BOLD-fMRI analysis of the contrast mTBI < HCs in TS condition (FWE corrected).

Brain Regions	Hemisphere	BA	Peak MNI Coordinates			Z-Value	Size (Voxels)
			x	y	z		
dCau	L	NA	−18	8	14	2.638	112
dlSFG	L	8	−18	10	50	3.005	177
	R	6, 8	20	−6	64	3.295	421
mSFG	R	91	8	46	42	3.201	154
IFG	R	44, 45	44	10	20	3.109	268
ACC	L	32	−10	40	12	3.549	210

mTBI, mild traumatic brain injury; HCs, healthy controls; FWE, family wise error; TS, task switching; MNI, Montreal Neurological Institute; L, left; R, right; dCau, dorsal caudate; dlSFG, dorsolateral Superior Frontal Gyrus; mSFG, medial Superior Frontal Gyrus; IFG, inferior frontal gyrus; ACC, anterior cingulate cortex.

## Data Availability

The data that support the findings of this study are available on request from the corresponding author.

## Supplementary Materials

The following supporting information can be downloaded at: https://www.mdpi.com/article/10.3390/jcm11092484/s1, Table S1: Results from the BOLD-fMRI analysis of the contrast mTBI < HC in OG condition (FEW corrected); Table S2: Results from the BOLD-fMRI analysis of the contrast mTBI < HC in DI condition (FWE corrected).
