# Supplementary material for: Abnormal Dorsal Caudate Activation Mediated Impaired Cognitive Flexibility in Mild Traumatic Brain Injury
Source: J Clin Med. 2022 Apr 28;11(9):2484. doi: 10.3390/jcm11092484 (PMC9105079; doi:10.3390/jcm11092484)
Supplement: Supplementary file 1 [file jcm-11-02484-s001.zip › jcm-1663911-supplementary.pdf]

Supplementary Materials

fMRI Behavioral Performance

Our data demonstrated that the patients with mTBI had significantly more ER (t_{31} = 3.098, $P < 0.005$) and longer RT (t_{31} = 1.638, $P > 0.05$) compared to HCs during the whole task.

In RT analyses, a 3 × 2 ANOVA with task condition (OG, DI, TS conditions) and group (mTBI patients, HCs) as independent variables revealed a significant main effect of task condition [$F_{(2,62)} = 174.655$, $P < 0.001$], with no task condition × group interaction [$F_{(2,62)} = 0.221$, $P = 0.802$] or main effect of group condition [$F_{(1,31)} = 3.847$, $P = 0.059$] (Figure 2A). Post hoc analyses revealed significantly higher RTs in the TS condition compared to the OG condition ($P < 0.001$) and the DI condition ($P < 0.001$) in both groups. In addition, there was significantly longer RTs for the DI condition compared with the OG condition ($P < 0.001$) in both groups. Furthermore, compared with HCs, patients with mTBI exhibited significantly longer RT in the TS condition [$F_{(1,31)} = 4.247$, $P = 0.048$], reflecting impaired cognitive flexibility on behavior level.

The ER showed that there were both a significant main effect of task condition [$F_{(2,62)} = 7.875$, $P < 0.001$] and main effect of group condition [$F_{(1,31)} = 9.953$, $P = 0.004$], without task condition × group interaction [$F_{(2,62)} = 2.639$, $P = 0.079$] (Figure 2B). Post hoc analyses demonstrated that ER was significantly higher for the TS condition as compared to the OG condition or the DI condition in mTBI patients ($P < 0.05$). There were no significant differences between all conditions in HCs [$F_{(2,62)} = 0.494$, $P = 0.615$]. Furthermore, the mTBI patients were significantly less accurate than HCs across all conditions ($P < 0.05$).

Table S1 Results from the BOLD-fMRI analysis of the contrast mTBI < HC in OG condition (FEW corrected)

Brain regions	Hemisphere	BA	Peak MNI coordinates			Z-value	Size(voxels)
			x	y	z		
mSFG	R	8, 9	10	20	50	2.804	152
mOG	R	14	4	30	-12	2.745	32
dlSFG	R	6, 8	20	26	42	2.936	309
vlMFG	R	6	28	0	50	2.703	180
rdSG	R	40	64	-30	46	3.375	126

mTBI, mild traumatic brain injury; HCs, healthy controls; FWE, family wise error; OG, ongoing; MNI, Montreal Neurological Institute; R, right; mSFG, medial Superior Frontal Gyrus; mOG, medial Orbital Gyrus; dlSFG, dorsolateral Superior Frontal Gyrus; vlMFG, ventrolateral Middle Frontal Gyrus; rdSG, rostrodorsal Supramarginal Gyrus.

Table S2 Results from the BOLD-fMRI analysis of the contrast mTBI < HC in DI condition (FWE corrected)

Brain regions	Hemisphere	BA	Peak MNI coordinates			Z-value	Size(voxels)
			x	y	z		
PCC	L	23	-12	-16	42	3.016	253
dlSFG	L	8	-16	22	40	2.359	217
	R	6, 8	20	30	40	3.214	368
mSFG	L	10	-10	60	6	3.219	353
	R	6, 9, 91	6	48	40	3.313	280
vlMFG	R	6	26	-2	46	2.957	171
pIns	R	NA	34	-10	14	3.442	109

mTBI, mild traumatic brain injury; HCs, healthy controls; FWE, family wise error; DI, distractor inhibition; MNI, Montreal Neurological Institute; L, left; R, right; PCC, posterior cingulate cortex; dlSFG, dorsolateral Superior Frontal Gyrus; mSFG, medial Superior Frontal Gyrus; vlMFG, ventrolateral Middle Frontal Gyrus; pIns, posterior insula.
